# A semantics-oriented computational approach to investigate microRNA regulation on glucocorticoid resistance in pediatric acute lymphoblastic leukemia

**DOI:** 10.1186/s12911-018-0637-3

**Published:** 2018-07-23

**Authors:** Huiqin Chen, Dihua Zhang, Guoping Zhang, Xiaofeng Li, Ying Liang, Mohan Vamsi Kasukurthi, Shengyu Li, Glen M. Borchert, Jingshan Huang

**Affiliations:** 1 Department of Pediatrics, The Third Affiliated Hospital, Sun Yat-sen University, Guangzhou, Guangdong China; 2 Department of Nephrology, The First Affiliated Hospital, Sun Yat-sen University, Guangzhou, Guangdong China; 3 Department of Oncology, Yuebei People’s Hospital, Shaoguan, Guangdong China; 4 School of Computing, University of South Alabama, Mobile, AL 36688 USA; 5 College of Medicine, University of South Alabama, Mobile, AL 36688 USA; 6 College of Math and Computer Science, Fuzhou University, Fuzhou, Fujian China; 7 School of Information, Qilu University of Technology (Shandong Academy of Sciences), Jinan, Shandong China

**Keywords:** Acute lymphoblastic leukemia (ALL), Drug resistance, microRNA (miRNA or miR), Glucocorticoids (GC), miRNA target, Biomedical and biological ontology (bio-ontology), Semantic integration, Semantic search

## Abstract

**Background:**

Acute lymphoblastic leukemia is the most prevalent neoplasia among children. Despite the tremendous achievements of state-of-the-art treatment strategies, drug resistance is still a major cause of chemotherapy failure leading to relapse in pediatric acute lymphoblastic leukemia. The underlying mechanisms of such phenomenon are not yet clear and subject to further exploration. Prior research has shown that microRNAs can act as post-transcriptional regulators of many genes related to drug resistance. However, details of microRNA regulation mechanisms in pediatric acute lymphoblastic leukemia are far from completely understood.

**Methods:**

We utilized a computational approach based upon emerging biomedical and biological ontologies and semantic technologies to investigate the important roles of microRNA: mRNA regulation on glucocorticoid resistance in pediatric acute lymphoblastic leukemia. In particular, various filtering mechanisms were designed based on the user-provided MeSH term to narrow down the most promising microRNAs in an effective manner.

**Results:**

During our manual search on background literature, we found a total of 18 candidate microRNAs that possibly regulate glucocorticoid resistance in pediatric acute lymphoblastic leukemia. After the first-round filtering using the Broader-Match option where both the user-provided MeSH term and its direct parent term were utilized, the number of targets for 18 microRNAs was reduced from 232 to 74. During the second-round filtering with the Exact-Match option where only the MeSH term itself was utilized, the number of targets was further reduced to 19. Finally, we conducted semantic searches in the OmniSearch software tool on the five likely regulating microRNAs and identified two most likely microRNAs.

**Conclusions:**

We successfully identified two microRNAs, hsa-miR-142-3p and hsa-miR-17-5p, which are computationally predicted to closely relate to glucocorticoid resistance, thus potentially serving as novel biomarkers and therapeutic targets in pediatric acute lymphoblastic leukemia.

## Background

Acute lymphoblastic leukemia (ALL) is the most common childhood malignant tumor and accounts for about 80–85% of acute leukemia in children. During the last four decades, great achievements have been made in ALL treatment, and the disease-free survival rates have increased to approximately 85% in developed countries [1–3]. Glucocorticoids (GCs) such as prednisone and dexamethasone (DEX) are essential drugs for ALL chemotherapy, and response to GC treatment is a strong independent factor of ALL prognosis. Currently, the 10-year event-free survival rate is about 80% for pediatric ALL patients with good response to prednisone. Unfortunately, there are still 57% of patients who have poor response to prednisone, and resistance to prednisone has become one of the main obstacles to achieve successful treatment outcomes in pediatric ALL [1–4]. As a result, there is an urgent need to investigate the mechanisms underlying GC resistance and thereby to explore novel therapeutic strategies to reverse GC resistance, which will help achieve full treatment potential and significantly improve prognosis.

Prior research [5, 6] showed that abnormal expression of microRNAs (miRNAs or miRs) is closely associated with GC resistance in pediatric ALL. Understanding miRNA regulation mechanisms in the modulation of GC response can potentially result in the discovery of novel molecular markers by which to predict GC resistance and thus to design therapeutic targets. Unfortunately, the exploration of miRNA: mRNA regulation is not trivial, which requires: (1) querying PubMed database [7] to look for biologically validated miRNA targets; (2) finding computationally putative miRNA targets by inquiring on numerous prediction databases such as miRDB [8], TargetScan [9], and miRanda [10]; and (3) obtaining additional information for each candidate target from data sources such as Gene Ontology (GO) [11] annotations, RNAcentral sequence annotations [12], pathway analysis by Protein ANalysis THrough Evolutionary Relationships (PANTHER) Classification System [13], the National Center for Biotechnology Information (NCBI) Gene database [14], and the Medical Headings (MeSH) database [15]. These data sources have heterogeneous semantics, and conventional, non-semantics-oriented knowledge discovery approaches are not suitable to properly handle complex semantics embedded in such big data. Therefore, it is critical to seek assistance from emerging semantic technologies, if more flexible, effective, and efficient miRNA knowledge acquisition is desired. In this paper, we introduce the utilization of OmniSearch software [16–18], a semantic search tool based upon biomedical and biological ontologies (bio-ontologies) and semantic technologies, to help better investigate miRNA regulation mechanisms in GC-resistant pediatric ALL. Note that our research efforts were initially reported in our BIBM 2017 proceeding paper [19]. Compared with the conference paper, this extended journal version has at least 75% new contents. To be more specific, all sections were significantly extended, detailed as follows. In “Background” Section, we added 17 papers into the background literature and gave a thorough discussion on our research background as well as related work. In “Methods” Section, we described all steps in our methods with significantly greater details. In particular, the manual search was described in detail along with 11 new references; and the MeSH-term filtering mechanisms were explained in detail along with a new figure. In “Results and Discussion” Section, we added greater details of results obtained from our methods, including detailed explanation and discussion in text, two new figures, and five new tables. In addition, we also conducted effectiveness and efficiency analysis in the OmniSearch software and reported these results. In “Conclusions” Section, we enriched it by adding details of our plan for future work.

## Methods

### Approach overview

We first performed a manual background literature search to identify an initial set of miRNAs that might be involved in the regulation of GC resistance in pediatric ALL. Next, we used the OmniSearch tool and user-provided MeSH terms to filter out more likely regulating miRNAs. Finally, we conducted semantic searches on this smaller set of miRNAs and obtained federated knowledge including putative and/or validated targets, associated GO annotations, related PubMed publications, RNAcentral sequence annotations, and pathway analysis. Based on such comprehensive search results, we disclosed the most promising miRNAs, along with their respective targets, as candidate novel biomarkers in GC-resistant pediatric ALL.

### Bio-ontologies underlying OmniSearch

**Fig. 1:**
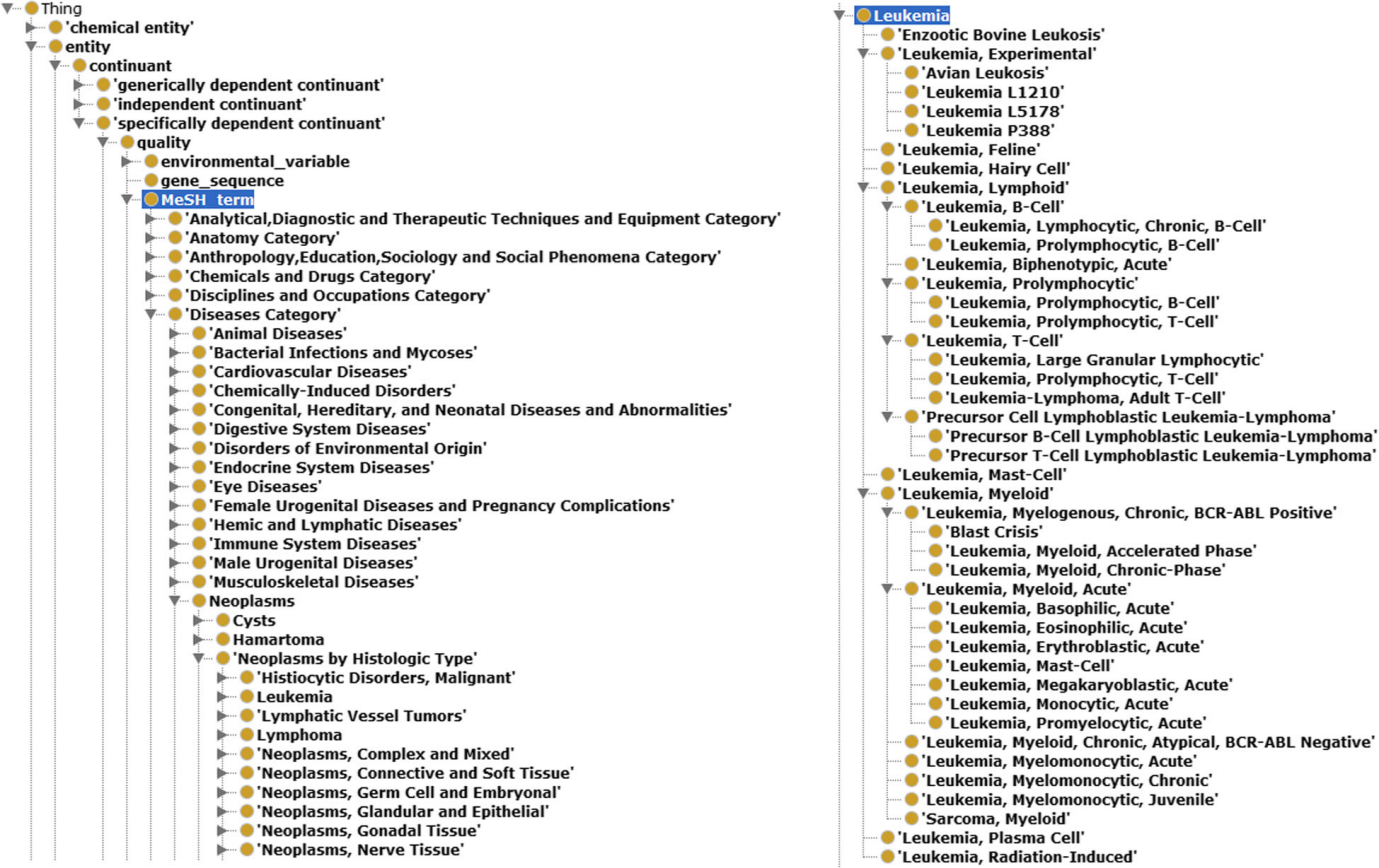
BFO-compliant, well-structured MeSH terms in the OMIT ontology presented in Protégé, an ontology development tool from Stanford. Left: the term MeSH term along with its direct parent, all ancestors, some siblings, and some offspring terms. Right: the entire set of offspring terms for the term *Leukemia*

Being a semantic integration & search software system, OmniSearch was built upon Ontology for MicroRNA Target (OMIT) [20] and Non-Coding RNA Ontology (NCRO) [21]. Technical details of these two Basic Formal Ontology (BFO)-compliant bio-ontologies were included in our previously published work [20, 21]. In this paper, we want to emphasize the well-structured MeSH terms defined in the OMIT ontology. As shown in Fig. 1: (1) all ontological terms are organized under the is_a hierarchy; (2) the left portion illustrates the term MeSH term along with its direct parent, all ancestors, some siblings, and some offspring terms; and (3) the right portion shows the entire set of offspring terms for the term *Leukemia*.

### Semantics-oriented MeSH-term filtering mechanisms

Technical details of the OmniSearch software tool were reported in our earlier work [16–18]. In this study, we have implemented user-friendly and highly flexible filtering mechanisms through user-provided MeSH terms. First of all, users can input any portion of a MeSH term that is related to their semantic search query. Depending on the user selection of “Match from the beginning” or “Match anywhere,” OmniSearch will return a list of relevant MeSH terms. This feature is user-friendly and convenient, especially to those users who are either not familiar with MeSH terms or not sure about the accurate spelling of a specific MeSH term.

Once a MeSH term is included into the user search query, there are three different filtering options: Exact-Match, Broader-Match, or Narrower-Match. The default selection is the Exact-Match option, where a record will be retrieved only if it contains the exact user-provided MeSH term. The other two filtering options are both based upon the semantics formally encoded in the OMIT ontology, detailed as follows.

Under the Broader-Match option, both the user-provided MeSH term and all of its parent terms will be used to filter out records. A record will be retrieved if it contains the MeSH term itself or any of its parent terms. For example, suppose the user supplies in the search query a MeSH term Leukemia, B-Cell, whose parent term is Leukemia, Lymphoid; all records containing either B-Cell leukemia or lymphoid leukemia will be returned. This option is useful in situations where the user-provided MeSH term is too specific (e.g., Leukemia, B-Cell), which will result in excluding records that are actually relevant (e.g., related to some lymphoid leukemia other than B-Cell leukemia) and should not be filtered out.

On the contrary, under the Narrower-Match option, both the user-provided MeSH term and all of its offspring terms will be utilized. A record will be retrieved if it contains the MeSH term itself or any of its offspring terms. For example, suppose the user supplies a MeSH term Leukemia, T-Cell, which has three offspring terms: Leukemia, Large Granular Lymphocytic; Leukemia, Prolymphocytic, T-Cell; and Leukemia-Lymphoma, Adult T-Cell; any records containing T-Cell leukemia or any of its subtypes will be returned. The motivation of having this option available is to deal with situations where the user-provided MeSH term is too general.

In summary, whereas the Exact-Match option is appropriate for users who are confident on their selected MeSH terms, both the Broader-Match and Narrower-Match options, when deemed necessary, are able to effectively expand original search queries and, in a user-friendly and highly flexible manner.

## Results

### Results from manual background literature search

During our manual search on background literature, we found a total of 18 candidate miRNAs that possibly regulate GC resistance in pediatric ALL: hsa-miR-124-3p [22], hsa-miR-124-5p [22], hsa-miR-128-3p [23], hsa-miR-142-3p [24], hsa-miR-15b-3p [25], hsa-miR-17-5p [26], hsa-miR-18a-3p [27], hsa-miR-182-5p [28], hsa-miR-193a-3p [27], hsa-miR-218-5p [27], hsa-miR-221-3p [29], hsa-miR-335-5p [30], hsa-miR-532-5p [27], hsa-miR-550a-3p [27], hsa-miR-625-5p [27], hsa-miR-633 [27], hsa-miR-638 [27], and hsa-miR-708-5p [31, 32]. GC response can be mediated by miRNAs through their influence on GC signaling pathway, leading to diverse GC responsiveness. On the other hand, GCs can regulate the function of some miRNAs, thus suggesting a bidirectional influence between GCs and miRNAs. We have reviewed and searched PubMed literature related to the effects of miRNAs on GC responsiveness in vivo or in vitro experiments, and the terms that we used in the manual search included: miRNAs, glucocorticoid response, glucocorticoid resistance, glucocorticoid receptors, and acute lymphoblastic leukemia. All the acquired miRNAs in the background literature search were selected as potential target miRNAs for further investigation.

### More likely regulating miRNAs through two rounds of the MeSH-term filtering

Before applying the MeSH-term filtering, a large number of targets (either computationally putative or biologically validated) were retrieved for each of the 18 miRNAs. As exhibited in Table 1, the total number of targets for these 18 miRNAs is 232; that is, more than 2 hundred miRNA: target pairs need to be further investigated, which would simply be, if not impossible, too time-consuming and labor-intensive.

**Table 1:** Number of targets retrieved in OmniSearch without applying the MeSH-term filtering

miRNA	Number of reporting databases	Number of total targets supported by at least one publication
hsa-miR-124-3p	3	15
hsa-miR-124-5p	2	0
hsa-miR-128-3p	3	5
hsa-miR-142-3p	4	69
hsa-miR-15b-3p	2	0
hsa-miR-17-5p	3	84
hsa-miR-18a-3p	3	4
hsa-miR-182-5p	3	16
hsa-miR-193a-3p	4	17
hsa-miR-218-5p	3	2
hsa-miR-221-3p	3	1
hsa-miR-335-5p	3	8
hsa-miR-532-5p	3	5
hsa-miR-550a-3p	2	0
hsa-miR-625-5p	2	1
hsa-miR-633	2	0
hsa-miR-638	2	3
hsa-miR-708-5p	3	2
Average	3	13
Total	–	232

As detailed in Section “Methods,” OmniSearch supplies users with flexible, semantics-oriented filtering on relevant MeSH terms. After the first-round filtering using the Broader-Match option where both the user-provided MeSH term (Leukemia in this study) and its direct parent term (Neoplasms by Histologic Type) were utilized, the number of targets for each miRNA was significantly reduced, as exhibited in Table 2 (note that the numbers of predicted and validated targets in both Table 2 ad Table 3 were obtained from the OmniSearch user interface). In particular, there are six miRNAs (hsa-miR-221-3p, hsa-miR-633, hsa-miR-550a-3p, hsa-miR-638, hsa-miR-124-5p, and hsa-miR-15b-3p, all italicized in the table) that had no retrieved targets after the filtering. That is, no putative and/or validated targets of these six miRNAs are yet discovered to be related to either leukemia or some other neoplasms of various histologic types. Therefore, it is less likely that these miRNAs have some regulation on GC resistance in pediatric ALL. Consequently, these miRNAs were removed from further consideration.

**Table 2:** Targets retrieved after applying the first-round MeSH-term filtering (broder-match on “leukemia”)

miRNA	Number of predicted targets	Number of validated targets
hsa-miR-142-3p	12	11
hsa-miR-17-5p	7	11
hsa-miR-18a-3p	2	2
hsa-miR-128-3p	4	0
hsa-miR-193a-3p	4	1
hsa-miR-124-3p	0	7
hsa-miR-182-5p	4	4
hsa-miR-335-5p	1	0
hsa-miR-708-5p	0	1
hsa-miR-625-5p	1	0
hsa-miR-218-5p	1	0
hsa-miR-532-5p	0	1
*hsa-miR-221-3p*	*0*	*0*
*hsa-miR-633*	*0*	*0*
*hsa-miR-550a-3p*	*0*	*0*
*hsa-miR-638*	*0*	*0*
*hsa-miR-124-5p*	*0*	*0*
*hsa-miR-15b-3p*	*0*	*0*
Total	36	38

**Table 3:** Targets retrieved after applying the second-round MeSH-term filtering (exact-match on “leukemia”)

miRNA	Number of predicted targets	Number of validated targets
hsa-miR-142-3p	5	4
hsa-miR-17-5p	3	3
hsa-miR-18a-3p	2	0
hsa-miR-128-3p	1	0
hsa-miR-193a-3p	0	1
*hsa-miR-124-3p*	*0*	*0*
*hsa-miR-182-5p*	*0*	*0*
*hsa-miR-335-5p*	*0*	*0*
*hsa-miR-708-5p*	*0*	*0*
*hsa-miR-625-5p*	*0*	*0*
*hsa-miR-218-5p*	*0*	*0*
*hsa-miR-532-5p*	*0*	*0*
Total	11	8

During the second-round filtering with the Exact-Match option where only the MeSH term Leukemia itself was utilized, the number of targets for each miRNA was further reduced, as shown in Table 3. Seven miRNAs (hsa-miR-124-3p, hsa-miR-182-5p, hsa-miR-335-5p, hsa-miR-708-5p, hsa-miR-625-5p, hsa-miR-218-5p, and hsa-miR-532-5p, all italicized in the table) had no retrieved targets. In other words, no putative and/or validated targets of these seven miRNAs are yet discovered to be directly related to leukemia even though they are related to some neoplasms other than leukemia. Therefore, these seven miRNAs were excluded from further analysis.

After two rounds of the MeSH-term filtering, we obtained a set of five likely regulating miRNAs: hsa-miR-142-3p, hsa-miR-17-5p, hsa-miR-18a-3p, hsa-miR-128-3p, and hsa-miR-193a-3p, along with a total of 19 respective targets, for further analysis. Note that if not for the effective filtering mechanisms provided in the OmniSearch software tool, we would have had to analyze 232 rather than 19 miRNA: target pairs. Up to this point, almost a 92% reduction in human efforts was already achieved. We will continue our discussion in Section “Discussion” on the efficiency feature in the OmniSearch software.

## Discussion

### Analysis of miRNA: Target regulation via semantic search

**Fig. 2:**
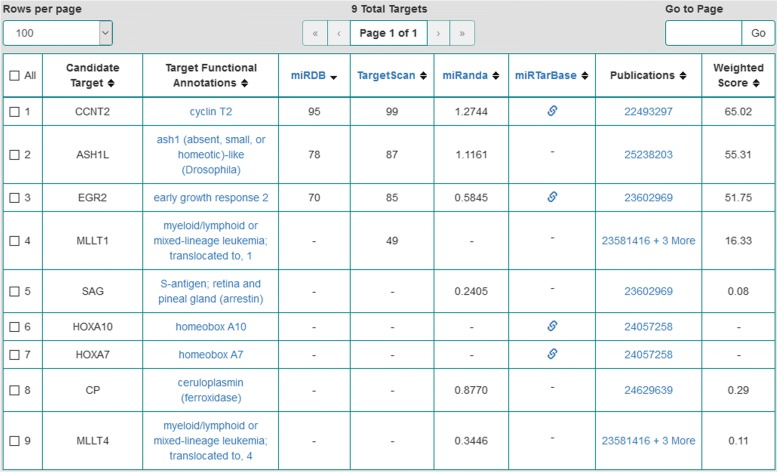
OmniSearch semantic search results for hsa-miR-142-3p (with the MeSH term *Leukemia* and the Exact-Match option)

**Fig. 3:**
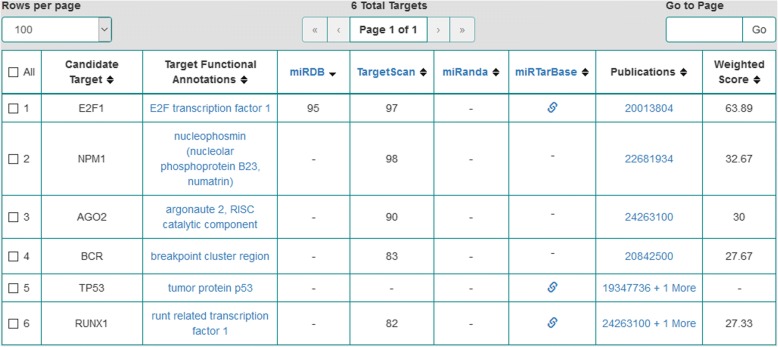
OmniSearch semantic search results for hsa-miR-17-5p (with the MeSH term *Leukemia* and the Exact-Match option)

We conducted semantic searches in the OmniSearch software tool on the five likely regulating miRNAs. Federated knowledge was obtained for each miRNA: (1) computationally predicted and/or biologically validated targets; (2) associated GO annotations; (3) supporting PubMed publications; (4) RNAcentral sequence annotations for involved ncRNA molecules; and (5) pathway analysis results on relevant targets. Table 4 lists both predicted and validated targets for these five miRNAs, where each target name is followed by its formal gene name (in square brackets). Figures 2 and 3 are screenshots of the results when conducting semantic searches on hsa-miR-142-3p and hsa-miR-17-5p, respectively. Screenshots for the other three miRNAs are not included due to the space limit.

**Table 4:** Putative and validated targets for each of the more likely regulating miRNAs

More likely regulating miRNAs	Computationally predicted targets	Biologically validated targets
hsa-miR-142-3p	• ASH1L [ash1 (absent, small, or homeotic)-like (Drosophila)] • CP [ceruloplasmin (ferroxidase)] • MLLT1 [myeloid/lymphoid or mixed-lineage leukemia; translocated to, 1] • MLLT4 [myeloid/lymphoid or mixed-lineage leukemia; translocated to, 4] • SAG [S-antigen; retina and pineal gland (arrestin)]	• CCNT2 [cyclin T2] • EGR2 [early growth response 2] • HOXA10 [homeobox A10] • HOXA7 [homeobox A7]
hsa-miR-17-5p	• AGO2 [argonaute 2, RISC catalytic component] • BCR [breakpoint cluster region] • NPM1 [nucleophosmin (nucleolar phosphoprotein B23, nunatrin)]	• E2F1 [E2F transcription factor 1] • RUNX1 [runt related transcription factor 1] • TP53 [tumor protein p53]
hsa-miR-18a-3p	• ARHGAP26 [Rho GTPase activating protein 26] • IMPACT [impact RWD domain protein]	None
hsa-miR-128-3p	• PHF6 [PHD finger protein 6]	None
hsa-miR-193a-3p	None	• MCL1 [myeloid cell leukemia 1]

We speculated that **hsa-miR-142-3p** and **hsa-miR-17-5p** are the two most promising miRNAs related to GC resistance in pediatric ALL. Our observations on comprehensive knowledge retrieved in OmniSearch are summarized below.

According to the research reported in [33], hsa-miR-142-3p was originally identified as a hematopoietic specific miRNA and expressed in various hematopoietic cell lineages. Lv M et al. [34] reported that T-cell ALL patients with high expression of hsa-miR-142-3p had a significantly shorter survival time than those with low expression. The poor prognosis was closely correlated to the oncogenic role of hsa-miR-142-3p that was mediated by decreasing CAMP levels, promoting T-leukemic cell growth, and inducing resistance to GC treatment through targeting GC receptor-α. To date, whether hsa-miR-142-3p is involved in B-cell ALL remains unknown. Ding S et al. confirmed that hsa-miR-142-3p was significantly down-regulated in SLE CD4+ T cells compared with healthy controls; in addition, the hsa-miR-142-3p level was inversely correlated with the putative SLE-related targets signaling lymphocytic activation molecule-associated protein (SAP), CD84, and interleukin-10 (IL-10). The reduced hsa-miR-142-3p expression in CD4+ T cells can significantly increase protein levels of these target genes and causes B cell hyperstimulation.

In 2012, Harada M et al. [26] examined the effect of DEX treatment on the miRNA profile of B-ALL cell lines and found the down-regulation of a group of miRNAs that were most notable members of the miR-17 cluster. Furthermore, experiments on primary ex vivo ALL cells found that apoptosis induced by DEX was related to down-regulated hsa-miR-17-5p levels. On the contrary, up-regulation of hsa-miR-17-5p resulted in a decrease in Bim protein levels and DEX-induced cell death. Therefore, hsa-miR-17-5p and other miR-17 members might play important roles in GC-induced cell death and GC resistance in B-ALL.

In addition, we further analyzed two targets of hsa-miR-142-3p: SAG [S-antigen; retina and pineal gland (arrestin)] and EGR2 [early growth response 2]. Arrestins (β-arrestin-1 and β-arrestin-2) are gene produts of SAG. The non-visual arrestins are multi-functional scaffolding proteins that play critical roles in G protein-coupled receptors (GPCRs) signaling. Upon binding activated GPCRs at the plasma membrane, β-arrestin terminates G protein-dependent responses (desensitization) and stimulates β-arrestin-dependent signaling pathways. Alteration of β-arrestin levels was confirmed in many human diseases, including Parkinson’s disease with dementia, multiple sclerosis, type 2 diabetes, coronary artery disease, and various cancers. However, very little is known about the β-arrestin levels and factors that control β-arrestin gene expression in ALL. GCs enhance the expression of β-arrestin-1 and repress the expression of β-arrestin-2 at the transcriptional level by the binding of GC receptor (GR) to intragenic GC response elements (GREs). The increased expression of β-arrestin-1 after GC treatment impairs G protein-dependent activation of inositol phosphate signaling, thereby enhancing β-arrestin-1-dependent stimulation of the MAPK pathway by protease-activated receptor 1. Despite all the aforementioned findings, whether SAG plays a role in the regulation of β-arrestin expression and GC resistance in childhood ALL still remains to be elucidated.

EGRs were initially identified as immediate early genes functioning as a convergence point from many stimuli such as growth factors, hormones, and neurotransmitters. EGR2 has been widely studied in hindbrain development; peripheral nerve myelination; cognitive processes; and T-cell activation, induction of anergy, and development. Interestingly, EGR2 was identified as a crucial mediator for epidermal growth factor (EGF)-induced osteoprogenitor proliferation and survival. In addition, EGR2 was also one of the most strongly inhibited genes in GC-treated MC3T3 cells. GC treatment leads to a drastic decrease in bone marrow osteoprogenitor number and an increase in osteoblast and osteocyte apoptosis in mice. Furthermore, EGR2-stimulated survival was due to increasing the amount of antiapoptotic protein MCL1. However, whether EGR2 is directly involved in regulating GC-resistance of childhood ALL is still not known.

Based on the above comprehensive analysis, it is reasonable to consider **hsa-miR-142-3p** and **hsa-miR-17-5p** as the two most likely regulating miRNAs in GC-resistant pediatric ALL. Therefore, these two miRNAs as well as their respective targets can potentially serve as novel biomarkers and therapeutic targets in pediatric ALL treatment.

### Effective and efficient analysis in OmniSearch

OmniSearch is highly effective and efficient to help users to obtain their desired search results.

1. During our previous investigation [16–18], both effectiveness and efficiency features of the OmniSearch software tool were demonstrated in an objective and unbiased manner when tested among domain experts from numerous institutions.
2. As discussed earlier in Section “Results,” after applying the MeSH-term filtering, we only needed to focus on 19 rather than 232 miRNA: target pairs, giving us a 91.8% reduction in human efforts.
3. We asked five domain experts to further test OmniSearch efficiency. These experts are the first five co-authors of this paper, who are clinical investigators in pediatric ALL and/or biomedical scientists in miRNA research. Once the set of five likely regulating miRNAs was obtained, these experts were advised to use two different approaches, i.e., their conventional search methods vs. the OmniSearch tool, to explore potential miRNA: target regulation mechanisms in pediatric ALL. Time spent in each approach was recorded for each expert and then averaged among all experts. Details of efficiency evaluation on searching all five miRNAs are provided in Table 5. The data in the table clearly demonstrate that efficiency in OmniSearch is satisfactory – a large portion of user time was saved on: (1) obtaining search results (67% on average); (2) conducting pathway analysis (57% on average); and (3) comparing results across four miRNA target databases (63% on average).

**Table 5:** OmniSearch efficiency analysis: saved time for end users

miRNA under search	Percentage of saved time on obtaining search results	Percentage of saved time on conducting pathway analysis	Percentage of saved time on comparing results across different target databases
hsa-miR-142-3p	69%	59%	57%
hsa-miR-17-5p	71%	53%	63%
hsa-miR-18a-3p	53%	63%	59%
hsa-miR-128-3p	68%	58%	70%
hsa-miR-193a-3p	73%	51%	64%
Average for five miRNAs	67%	57%	63%

## Conclusions

ALL is the most important neoplasia among children. Although great progress has been made in clinical treatment during the past decades, drug resistance still remains a major cause of chemotherapy failure and relapse in pediatric patients. In particular, GC resistance is a critical clinical problem causing treatment failure in pediatric ALL patients. Therefore, there exists an urgent need to further understand the mechanisms of GC resistance and, in turn, to explore novel therapeutic strategies to improve treatment outcomes and disease prognosis. Overwhelming evidence illustrates that miRNAs act as post-transcriptional regulators of genes related to drug resistance, and deregulated miRNA expression might contribute to different GC treatment response in ALL. However, functions of individual miRNAs and their potential mechanisms involved in GC response are still largely unknown. In the present study, we introduced the utilization of OmniSearch, a semantic integration and search software tool, to facilitate the exploration of important roles of miRNAs performed on GC-resistant pediatric ALL. Our methodologies were demonstrated to be promising to significantly assist with unraveling miRNA regulation mechanisms. Specifically, the OmniSearch software is user-friendly, effective, and highly efficient. Using OmniSearch, we successfully disclosed two miRNAs, hsa-miR-142-3p and hsa-miR-17-5p, which likely regulate GC resistance in pediatric ALL. These two miRNAs as well as their respective targets can potentially serve as novel biomarkers and therapeutic targets.

An immediate piece of future work is to conduct wet-lab validation of biological functions of miRNAs along with their putative targets reported in this paper. We plan to perform luciferase reporter assays to confirm the suppression of target mRNAs by their paired miRNAs, followed by soft agar assays to assess the role of these miRNA: mRNA pairs in the regulation of GC resistance in pediatric ALL. Such wet-lab validation is supposed to improve the whole research pipeline and to reduce type I and II errors as well. Another promising research direction is to use semantic data mining techniques to explore both direct and indirect data associations among originally heterogeneous data sources. Such data associations, especially those indirect ones, will supply us with additional clues in further understanding miRNA regulation in pediatric ALL.

## Abbreviations

ALL: Acute lymphoblastic leukemia
BFO: Basic formal ontology
bio-ontologies: Biomedical and biological ontologies
DEX: Dexamethasone
GC: Glucocorticoid
GO: Gene ontology
GPCRs: G protein-coupled receptors
GR: GC receptor
GREs: GC response elements
IL-10: Interleukin-10
MeSH: Medical Headings
miRNA/miR: microRNA
NCBI: National Center for Biotechnology Information
NCI: National Cancer Institute
NCRO: Non-Coding RNA Ontology
NIH: National Institutes of Health
OMIT: Ontology for MicroRNA Target
PANTHER: Protein ANalysis THrough Evolutionary Relationships
SAP: SLE-related targets signaling lymphocytic activation molecule-associated protein
